# How does the environment in and around the home impact social care and health outcomes for older people?

**DOI:** 10.23889/ijpds.v8i2.2333

**Published:** 2023-09-14

**Authors:** William Midgley, Amy Mizen, Joe Hollinghurst, Robyn Hollinghurst, Ronan Lyons, Richard Fry

**Affiliations:** 1 Swansea University, Swansea, United Kingdom

Reducing the burden of falls and fall-related admissions to hospital and care homes is an important policy area. Falls cause significant injury leading to a reduced quality of life. We wanted to know if the environment around people’s homes changes the risk of falls for older people in Wales.

We linked routinely collected, anonymised health data on frailty, dementia, hospital admissions, care home admission, and A&E attendance in the Secure Anonymised Information Linkage Databank. We also linked individual level demographic data and household level neighbourhood walkability and accessibility metrics detailing the built environment (e.g. access to services, greenspace). We capture change in the built environment and house moves between January 2010 and December 2017. Using unadjusted and adjusted cox regression models we will assess how the risk and severity of a fall changes in relation to the built environment.

We have created a dynamic national e-cohort linking data on frailty, dementia, hospital admissions, care home admission, A&E attendance and built environment measures for people living in Wales aged 60 and above between January 2010 and December 2017. At the conference we will report summary statistics for each quarter as well as the overall population. We will also report unadjusted cox model odds ratios, as well as fully adjusted models. We will adjust for age, sex, urbanicity (urban/rural), deprivation (WIMD), dementia diagnosis, and seasonal weather trends. We will also report odds ratios for our stratification analysis where we will investigate whether associations vary by urbanicity and deprivation.

With an aging population, it is becoming more important to help the current and future older population age healthily, preventing adverse health outcomes before they happen. This research will help guide policy and resource allocation to support people staying at home and have a more fulfilling life for longer.

